# It’s All in the Eyes: Subcortical and Cortical Activation during Grotesqueness Perception in Autism

**DOI:** 10.1371/journal.pone.0054313

**Published:** 2013-01-14

**Authors:** Nicole R. Zürcher, Nick Donnelly, Ophélie Rogier, Britt Russo, Loyse Hippolyte, Julie Hadwin, Eric Lemonnier, Nouchine Hadjikhani

**Affiliations:** 1 Brain Mind Institute, EPFL, Lausanne, Switzerland; 2 School of Psychology, University of Southampton, Southampton, United Kingdom; 3 Centre de Ressources Autisme de Bretagne, CHRU Brest Hôpital Bohars, Bohars, France; 4 Université de Brest, CHRU Brest Hôpital Bohars, Bohars, France; 5 MGH-HMS-MIT A. Martinos Center for Biomedical Imaging, Harvard Medical School, Charlestown, Massachusetts, United States of America; Lyon Neuroscience Research Center, France

## Abstract

Atypical face processing plays a key role in social interaction difficulties encountered by individuals with autism. In the current fMRI study, the Thatcher illusion was used to investigate several aspects of face processing in 20 young adults with high-functioning autism spectrum disorder (ASD) and 20 matched neurotypical controls. “Thatcherized” stimuli were modified at either the eyes or the mouth and participants discriminated between pairs of faces while cued to attend to either of these features in upright and inverted orientation. Behavioral data confirmed sensitivity to the illusion and intact configural processing in ASD. Directing attention towards the eyes vs. the mouth in upright faces in ASD led to (1) improved discrimination accuracy; (2) increased activation in areas involved in social and emotional processing; (3) increased activation in subcortical face-processing areas. Our findings show that when explicitly cued to attend to the eyes, activation of cortical areas involved in face processing, including its social and emotional aspects, can be enhanced in autism. This suggests that impairments in face processing in autism may be caused by a deficit in social attention, and that giving specific cues to attend to the eye-region when performing behavioral therapies aimed at improving social skills may result in a better outcome.

## Introduction

Autism spectrum disorders (ASD) are neurodevelopmental disorders affecting close to 1% of the population, that are characterized by three behaviorally defined symptoms: impaired social interaction, deficits in communication and restrictive and repetitive behavior [1]. Decreased attention to faces, difficulties in reading facial expressions and emotions, failure to orient towards the eye region of the face and difficulties in understanding eye gaze have been reported in numerous studies (e.g. [2], [3], [4]). These aspects are determinant elements in diagnosis of ASD (e.g. [2], [5], [6]). Typical face perception is based on configural processing, which refers to the sensitivity of the spacing between features of a face, such as eyes and mouth. Those relations, commonly referred to as second-order relations [7], are automatically computed for typical upright faces. Inversion interferes with configural processing and inverted faces are processed using a feature-based strategy (e.g. [8], [9]).

In ASD, there has been a debate whether typical upright faces are processed configurally (e.g. [10], [11]) or using a feature-based strategy [12], [13]. A recent review of behavioral studies in face processing in ASD has concluded that face identity processing is qualitatively similar between people with ASD and individuals with neurotypical development, but that people with ASD have specific deficits discriminating the eyes during face processing [14].

One of the behavioral paradigms thought of as providing support for configural processing of faces is the Thatcher Illusion (TI). In the TI the eyes and mouth are inverted relative to the rest of the face [15]. When thatcherized faces are presented upright, they appear weird and grotesque, whereas this effect vanishes when they are presented inverted. The relationship between the TI and configural processing has been the subject of investigation [8], [16], [17], [18]. Recent studies have confirmed that configural processing is present in typical upright faces, as well as in upright faces which have been thatcherized at only one feature [19]. In contrast, the role of configural processing in fully thatcherized faces is unclear [19], [20]. Furthermore, we have recently shown that the efficacy of the illusion relies on a network of areas involved in social and emotional processing and which are engaged in mentalizing, including the medial prefrontal (mPFC)/orbitofrontal cortex and the posterior cingulate/precuneus. Discrimination between a typical face and a thatcherized face led to increased activation in the face-processing network when the faces were presented inverted [21]. Studies investigating face processing in normal inverted faces have yielded discrepant results. The face inversion effect has been specifically associated with decreased activation for inverted faces in the fusiform face area (FFA) [22] but also with increased activation in the object responsive lateral occipital cortex [23], [24].

Our previous work in a neurotypical population demonstrated the pre-eminent role of the eyes in generating the TI [21]. When looking at faces, adults with neurotypical development have a natural tendency to attend more to the eye region [25], and this is not the case in individuals with ASD [2], [3], [4]. There is evidence that people with ASD, rather than having non-specific difficulties in face processing, are specifically impaired with the processing of the eyes [26], [27]. To our knowledge, no fMRI study has so far addressed the contribution of the different features (eyes and mouth) to the TI in ASD. The current study employed thatcherized stimuli modified to tease apart the relative contribution of different facial features to the TI to further examine the neural substrate of face processing in individuals with ASD. Previous studies have shown that cueing to the eyes can improve performance in a configural face processing paradigm [11] and elicit typical brain activation in areas associated with face processing in individuals with ASD (e.g. [4], [11], [28]). Given that the eyes have been demonstrated to play a primary role in driving the TI [21], we hypothesized that cueing to the eyes would increase the sensitivity to the TI and therefore lead to heightened discrimination accuracy as well as to increased activation in cortical areas involved in social and emotional processing in participants with ASD.

Individuals with ASD have a natural tendency to avoid looking at the eyes and experimental designs requiring them to look at the eye region have led to increased amygdala activation [4], [29]. Together with the superior colliculus and the thalamus, the amygdala belongs to the subcortical extrageniculostriate route involved in rapid face detection. Given the use of cues to attend to the eye region in the current TI paradigm, we hypothesized that participants with ASD would show increased activation in this subcortical route.

In summary, three hypotheses were tested in this study: Directing visual attention towards the eyes in a TI discrimination task, leads to (1) better behavioral performance (2) increased activation in cortical areas involved in social and emotional processing and (3) increased activation in subcortical areas in individuals with ASD.

## Materials and Methods

### Participants

Twenty neurotypical controls (NT) and 20 normally intelligent individuals with ASD were enrolled in the study. All participants had normal or corrected to normal vision. Two NT and 4 ASD had to be excluded due to excessive movement during data acquisition. Sixteen participants with ASD (3 females, 23.5 years ±6.8 (mean ± SD)) and 18 NT participants (2 females, 25.8 years ±5.3) were included in the data analysis. Performance intelligence quotient (PIQ) was assessed using the Wechsler Non-verbal Scale or the Wechsler Abbreviated Scale of Intelligence [30], [31] and all participants had a PIQ in the normal range. Scores on the first series of the Raven’s Progressive Matrices Advanced were also obtained [32]. Groups were matched for age, PIQ and Raven’s score.

Participants with ASD were assessed by experienced clinicians on the Autism Diagnostic Observation Schedule (ADOS) and on the Autism Diagnostic Interview-Revised (ADI-R) [5], [6]. Seven had a diagnosis of Autism, 7 of Asperger’s syndrome and 2 were in the broad spectrum – Pervasive Developmental Disorder not otherwise specified (PDD-NOS). See Table 1 for participants’ characteristics.

**Table 1:** Participant characteristics.

	ASD	NT
**N number**	16	18
**Age, years**	23.5 (6.8)	25.8 (5.3)
**Non-verbal reasoning**		
PIQ	108.7 (13.3)	112.1 (9.0)
Raven's matrices	10.3 (1.9)	10.5 (1.0)
**ADI-R**		
Social	20.67 (3.94)	N/A
Communication	12.93 (4.20)	N/A
Stereotypies	4.27 (1.83)	N/A
Development	2.93 (1.44)	N/A
**ADOS**		
Communication	4.00 (1.37)	N/A
Social	7.88 (2.47)	N/A

Note: Data are presented as the mean and standard deviation in parentheses. Abbreviations: PIQ = Performance IQ, ADI-R = Autism Diagnostic Interview - Revised, ADOS = Autism Diagnostic Observation Schedule, N/A = not applicable.

**Figure 1:**
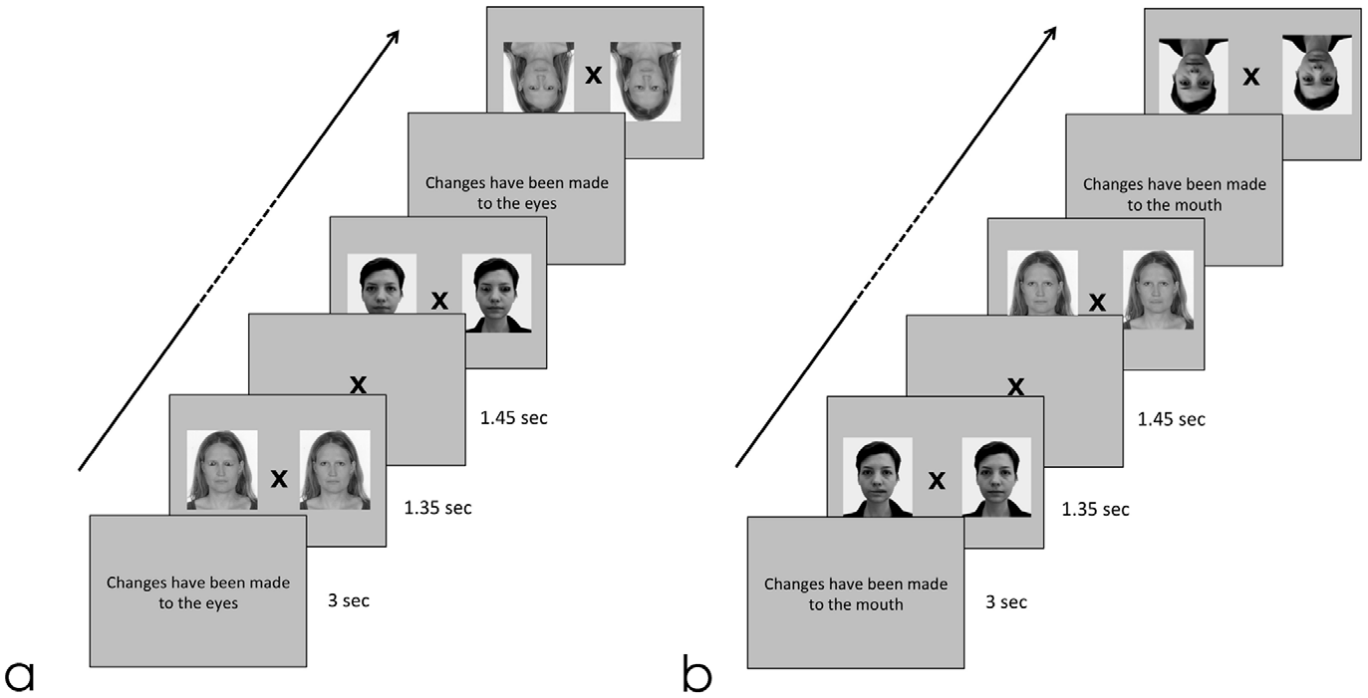
Example of the stimuli presented. Panel a: discrimination of stimuli thatcherized at the eye region. Panel b: discrimination of stimuli thatcherized at the mouth. Stimuli were presented in upright and inverted orientation for both conditions. Before each block, a cue indicated the location of thatcherization. Participants had to indicate with a button box whether the left or the right stimulus had been thatcherized. Note that those pictures do not represent the original identities used in the study.

The Lausanne University Hospital Ethical Committee approved the protocol and all procedures followed the Declaration of Helsinki. None of the participants were compromised in their capacity to assent/consent, and each of them, or their legal guardian for two minor participants, provided written informed consent after complete description of the study. The subjects in the photograph in Figure 1 gave written informed consent, as outlined in the PLOS consent form, to publication of their photograph.

### Behavioral Assessment

In addition to the ADOS and the ADI-R diagnostic tests, and in order to quantify the presence of autism traits, all participants completed the Autism Quotient (AQ) and Empathy Quotient (EQ) self-report questionnaires [27], [33]. Student *t*-tests were conducted to assess differences between groups.

### Stimuli

The stimuli used have been described in detail in previous studies [21], [34]. Sixteen identities were used. Thatcherized faces were paired with the non-thatcherized versions of the same faces, to create three types of stimulus pairs (face with thatcherized eyes vs. typical face, face with thatcherized mouth vs. typical face, and both features thatcherized vs. typical face) for each identity. It is important to note that the discriminability of the features used in this study (eyes and mouth) has been shown to be equal when the features were presented in isolation with no face contexts [35].

### Task Paradigm Used during fMRI (see Figure 1)

Visual stimuli, presented using the E-Prime software package (Psychological Software Tools, Pittsburgh, PA), were back-projected onto a screen positioned at the head of the scanner bore and viewed by the participants through an oblique mirror mounted on the head coil. The experiment was composed of two runs, each consisting of 16 blocks. Runs consisted of a single feature condition (eyes or mouth) alternating with the double feature condition. The sequence of the presentation of the two runs was counterbalanced across participants. A 3 second visual cue preceded each block and stated, “changes have been made to the eyes”, “changes have been made to the mouth” or “changes have been made to the eyes and mouth”. Each stimulus pair (modified face and its typical version) was presented for 1′350 ms during which participants responded. A fixation cross was then presented for 1′650 ms. Pairs of faces were presented in upright and inverted orientation, counterbalanced across blocks. Presentation of the target was counterbalanced between the left and the right side of the screen. Participants were told to press the button corresponding to the side of the location of the thatcherized stimulus. A button box was used to record participants’ responses to the stimuli. Behavioral data for two NT participants were lost due to a technical problem.

The main aim of the current study was to investigate the relative contribution of the eyes and the mouth to the TI in ASD; the double feature (modification to eyes and mouth) was also included in the experimental paradigm but does not represent the contrast of interest for the current study. In addition, double feature condition contrasts have to be interpreted with caution, because the cues given to look at the eyes or the mouth were found to have long lasting effects.

### fMRI Data Acquisition

Anatomical and functional MR images of brain activity were collected in a 3T high-speed echoplanar-imaging device (Tim Trio, Siemens, Erlangen) using a 12-channel matrix coil. Participants lay on a padded scanner couch and wore foam earplugs. Foam padding stabilized the head. High-resolution (1.0×1.0×1.0 mm^3^) structural images were obtained at the beginning of the session with a multi-echo magnetization-prepared rapid acquisition gradient echo (ME-MPRAGE) sequence (176 slices, FOV = 256, 256×256 matrix, echo time (TE1) = 1.64 ms, (TE2) = 3.5 ms, (TE3) = 5.36 (TE4) = 7.22 ms; repetition time (TR) = 2530 ms; flip angle = 7°. The co-registered functional acquisition (45 AC-PC slices, FOV = 216, matrix = 64×64, TE = 30 ms, TR = 3,000 ms, 3 mm thick, 3.12 mm by 3.12 mm in-plane resolution, flip angle 90°) lasted 384 seconds. A separate face and object functional localizer run was also obtained in all participants. The localizer scan consisted of alternating blocks of upright faces and objects [36] during which participants had to perform a one-back task.

### fMRI Data Analysis

FSL (FMRIB Software Library) package and techniques were used in data preprocessing and analysis. Specifically, FSL Brain Extraction Tool (BET) was used to remove non-brain tissue [37] and fMRI data processing was performed using FEAT (FMRI Expert Analysis Tool) version 5.98. [38], [39], [40]. Each functional run was first motion-corrected with MCFLIRT [41] and spatially smoothed with full width at half maximum of 8 mm. First-level analysis was performed using FILM (FMRIB’s Improved Linear Model), which uses a nonparametric estimation of time series autocorrelation to pre-whiten each voxel’s time series [42]. High pass temporal filtering with sigma = 50.0 s was applied to remove low frequency artifacts. Registration to high-resolution structural images was carried out using FMRIB’s linear registration tool (FLIRT) [41] and registration to standard space was further refined using FMRIB’s nonlinear registration tool (FNIRT, http://www.fmrib.ox.ac.uk/fsl/fnirt/index.html). To examine the TI effect, contrasts were conducted between upright faces (involving configural processing and grotesqueness perception) and inverted faces (involving featural processing) for each single feature condition. Mixed effects GLM analyses were carried out across participants using the two stages of FLAME (FMRIB’s Local Analysis of Mixed Effects) [43], [44], [45], an analysis allowing inference about the population from which the subjects were drawn. Threshold significance in the whole brain analysis for the within group data was *p _FDR_* <0.05, corrected for multiple comparisons using false discovery rate (FDR). Activation between groups was compared using a two sample unpaired *t*-test available in FSL. Statistical maps were thresholded using clusters determined by Z>2.3 and a corrected cluster significance threshold of *p = *0.05 [40].

### ROI Analyses

Regions of interest (ROIs) comprised cortical and subcortical areas involved in face and face inversion processing. The cortical ROIs comprised the fusiform face area (FFA), the lateral occipital cortex (LOC) and the pars opercularis of the inferior frontal gyrus (IFG) previously shown to be activated for discrimination of inverted thatcherized faces [21]. Subcortical ROIs consisted of the pulvinar nucleus of the thalamus (PUL) and the amygdala (AMY), both involved in rapid face detection. To avoid circularity, ROIs were defined by anatomical constraints or by independent functional constraints. The AMY and IFG were specified by labels corresponding to the 25% probability cortical and subcortical Harvard-Oxford atlases. The PUL was defined within the thalamic mask of the 25% probability Harvard-Oxford subcortical atlas, following anatomical landmarks [46]. Anatomical ROIs were then mapped back to each participant. An independent functional experiment with faces and objects was performed to define the functional ROIs for the FFA and LOC at the subject level. As there is strong evidence for right hemispheric dominance in face processing (e.g. [47], [48]), cortical ROIs were restricted to the right hemisphere. Subsequently, for each ROI, the percentage BOLD signal change was extracted from the mean (for all subcortical ROIs) or from the peak (for all cortical ROIs) of the parameter estimate at the subject-level for the contrasts of interest using FSL’s Featquery. A one-sample *t*-test against zero was conducted in order to determine whether the percent signal change for the contrast across orientation (upright vs. inverted) was significantly different from zero, indicating that there was increased activation for one or the other Orientation. Effects of Feature (eyes vs. mouth), Group (ASD vs. NT) and Feature x Group interactions were assessed with ANOVAs.

## Results

### Behavioral Assessment Questionnaires

ASD participants had an AQ score of 30.4±4.6 (mean ± SD) and an EQ score of 25.8±6.7. NT scored significantly lower on the AQ (*t*(32) = 9.58, *p*<0.001) and significantly higher on the EQ (*t*(32) = 4.78, *p*<0.001) with mean scores of 14.6±5.0 and 39.6±9.6 respectively.

### Behavioral Performance during the Thatcher Illusion Discrimination Task (Figure 2)

**Figure 2:**
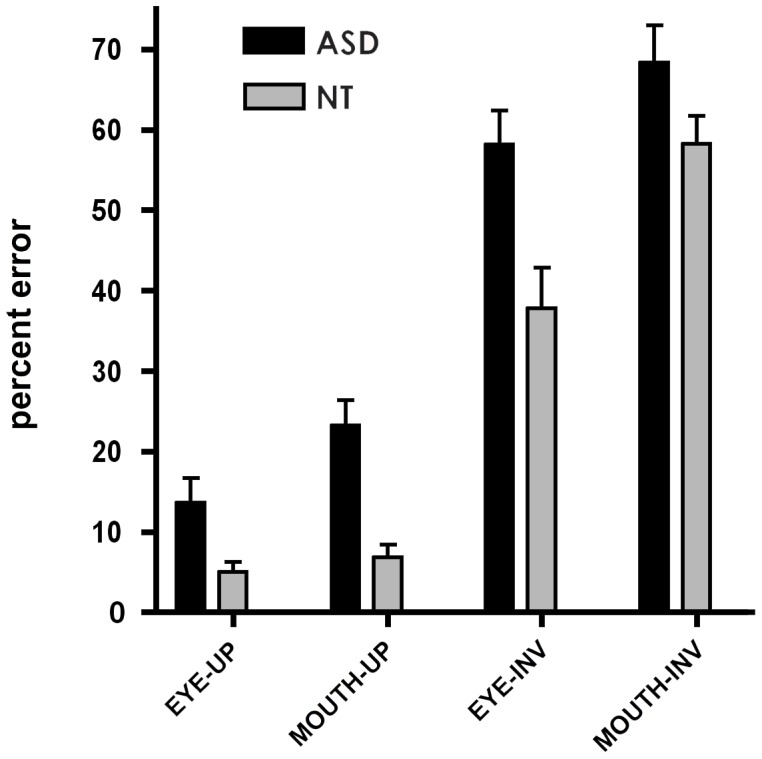
Behavioral results for Thatcher discrimination. Percentage error rates (with standard errors) across the different conditions for the behavioral Thatcher experiment. UP stands for stimuli presented in upright orientation, INV for those presented in inverted orientation. For both groups and all feature conditions, participants made significantly more errors for the inverted than for the upright condition *(p*<0.0001), reflecting sensitivity to the TI in both ASD and NT.

To assess how efficient participants were at discriminating thatcherized stimuli, we analyzed error rates, indicating wrong choice or omission, as well as reaction times. Error rates were analyzed in an ANOVA repeated over Feature (eyes vs. mouth) and Orientation (upright vs. inverted) with Group as the between-subject factor. As predicted, there was a significant Orientation effect (*F(1,30) = 259.43, p*<0.001, partial eta-squared (*η*_p_^2^) = 0.90) and no Orientation x Group interaction (*F*(1,30) = 0.26, *ns,* (*η*_p_^2^) = 0.009). Follow-up t-tests confirmed that both groups showed the orientation effect for both features (all *p*<0.05), demonstrating the presence of the Thatcher Illusion (grotesqueness detected in upright orientation but not inverted) in both ASD and NT. The interaction between Feature, Orientation and Group was significant (*F*(1,30) = 7.40, *p* = 0.01, *η*_p_^2^ = 0.20). Follow-up *t*-tests demonstrated that for NT error rates did not differ between eyes and mouth in upright orientation (eyes: (mean ± SEM) 5.0±1.3, mouth: 6.8±1.6, *ns*), while in inverted orientation they made more errors when cued to the mouth (eyes: 37.8±5.0, mouth: 58.3±3.4, *p*<0.05). ASD on the other hand, made fewer errors when cued to the eyes compared to when cued to the mouth in upright orientation (eyes: 13.6±3.1, mouth: 23.2±3.2, *p*<0.05) but only a trend for better discrimination of eyes compared to mouth in inverted presentation (eyes: 58.2±4.2, mouth: 68.4±4.6, *p* = 0.06). Moreover, NT showed higher accuracy than ASD for all conditions (all *p*<0.05) apart for the condition in which discrimination was made based on the mouth in inverted thatcherized faces *(see* *Figure 2* *).* Reaction times were analyzed in an ANOVA repeated over Feature (eyes vs. mouth) and Orientation of context (upright vs. inverted) with Group as the between-subject factor. A significant Orientation x Group effect (*F*(1,30) = 8.17, *p*<0.01, (*η*_p_^2^) = 0.21) was found. Follow up *t*-tests showed that this was due to faster reaction times in ASD for the inverted condition (NT upright (mean ± SEM): 840 ms ±15, ASD upright: 844 ms ±16, NT inverted: 886 ms ±25; ASD inverted: 690 ms ±56, *p*<0.01).

### Within-group Whole Brain Activation, for ASD and NT

#### Attending to the eyes (see figures 3 and 4, left panels, table 2)

In ASD only, attending to the eyes in upright faces resulted in activation in the subcortical route, amygdala, thalamus pulvinar, and superior colliculus as well as in the hippocampus and the anterior cingulate. For both groups, attending to the eyes in upright faces lead to significant activation in emotion processing and mentalizing areas (mPFC, orbitofrontal cortex, posterior cingulate cortex/precuneus cortex, posterior insula; *see activation in yellow)*, whereas attending to inverted faces lead to significant activation in extrastriate visual areas associated with face and object processing (fusiform gyrus, inferior occipital gyrus, lateral occipital cortex*; see activation in blue)*. NT in addition showed activation in the cerebellum, pallidum and in motor regions of the thalamus.

**Figure 3:**
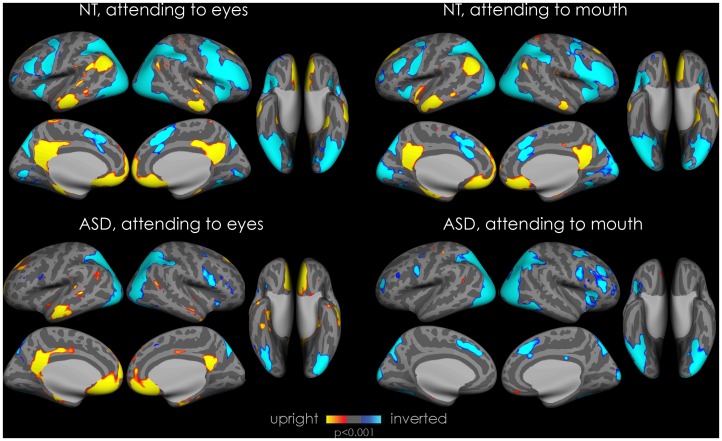
Cortical activation for within-group whole brain analysis. Statistical maps of differences in fMRI activation for each group for each condition. Statistical maps are displayed on the inflated cortical surface of the template FreeSurfer brain (fsaverage), at *p*<0.001 uncorrected, for visualization purposes, on the lateral, medial and ventral views of both hemispheres. Regions of greater activation for discrimination between upright thatcherized and normal faces are depicted in yellow to red; those for discrimination of inverted thatcherized faces from normal faces are depicted in cyan to blue. The grey mask covers subcortical regions in which activity cannot be expressed in surface rendering. The two left panels show activation for the condition where participants are attending to the eye-region to perform the task (top panel: NT; bottom panel: ASD). The two right panels show activation for the condition where participants are attending to the mouth-region to perform the task (top panel: NT; bottom panel: ASD).

**Figure 4:**
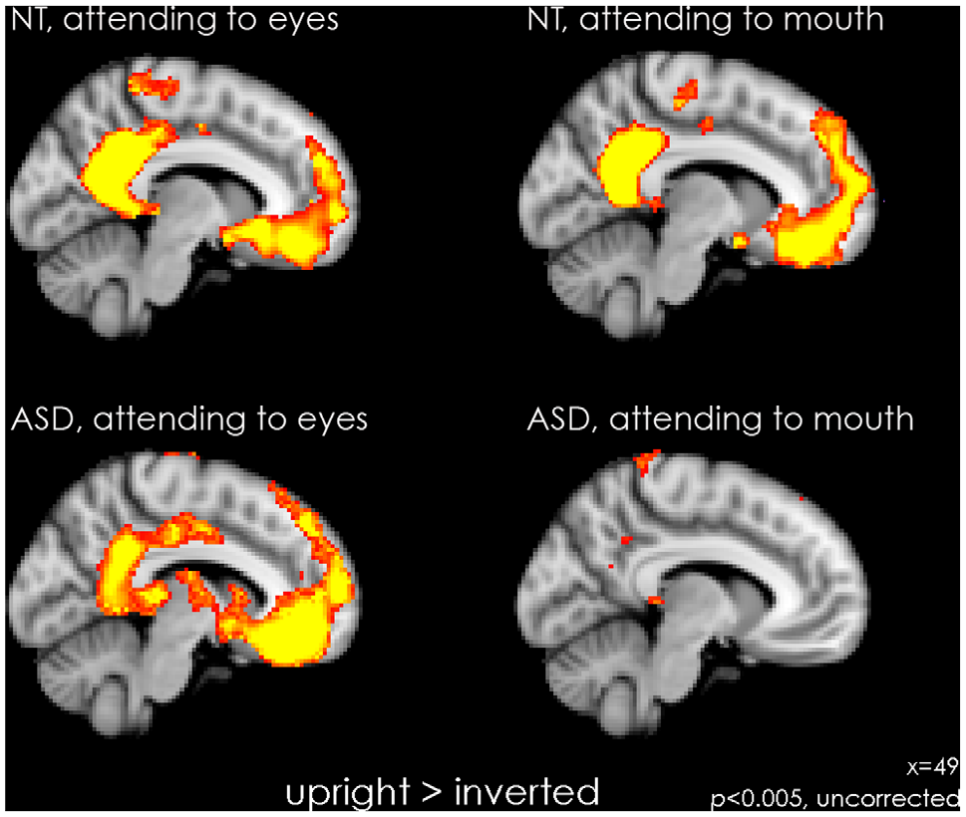
Cortical and subcortical activation for within-group whole brain analysis. Statistical maps of increased activation for each group for the contrast upright vs. inverted, showing areas of subcortical activation, displayed on the FSL MNI template at a sagittal slice x = 49. The two left panels show activation for the condition where participants are attending to the eye-region to perform the task (top panel: NT; bottom panel: ASD). The two right panels show activation for the condition where participants are attending to the mouth-region to perform the task (top panel: NT; bottom panel: ASD). Data are thresholded with *p*<0.005, uncorrected, for visualization purposes. When cued to the eyes, both groups showed activation in medial prefrontal cortex and posterior cingulate/precuneus cortex. In addition, ASD showed activation in subcortical structures. When cued to the mouth ASD do not show activation in medial prefrontal cortex and posterior cingulate/precuneus cortex.

**Table 2:** Within-group contrasts when participants are attending to the eyes and mouth, for upright and inverted conditions *p _FDR_ <*0.05.

	EYES UP									MOUTH UP							
		ASD				CON				ASD				CON			
Brain region	Hemi	x	y	z	Z value	x	y	z	Z value	x	y	z	Z value	x	y	z	Z value
amydala	LH	−24	−4	−24	3.51									−30	−2	−20	4.9
	RH													28	−6	−16	3.18
thalamus pulvinar	RH	12	−30	6	3.03												
	LH	−10	−30	6	4.15												
superior colliculus	LH	−6	−32	−2	2.9												
anterior cingulate	RH	4	44	−2	5.54												
	LH	−2	44	−2	5.45												
hippocampus	RH													22	−10	−24	3.81
	LH	−30	−14	−18	4.4									−24	−12	−20	6.58
medial prefrontal cortex	RH	2	36	−16	4.57	4	36	−16	4.58					2	46	−16	8.98
	LH	−6	36	−16	4.76	−4	36	−16	4.6					−4	38	−24	7.93
Subcallosal cortex	RH	2	22	−8	5.33	6	12	−10	5.75					2	30	−18	6.24
	LH	−8	30	−22	6.16	0	8	−10	5.38					−2	30	−18	7.27
Orbitofrontal cortex	LH	−32	28	−18	4.04	−28	32	−20	3.9					−28	30	−18	4.02
Posterior cingulate	RH	4	−46	34	4.01	2	−30	36	6.11					4	−26	40	4.08
	LH	−2	−46	34	4.63	−6	−32	40	4.56					−2	−52	24	9.83
Precuneus	RH	−6	−52	16	4.07	10	−56	26	8.23					0	−58	22	9.02
	LH	6	−52	18	4.21	−2	−58	36	6.67					0	−58	22	9.02
middle temporal gyrus posterior	RH	50	−14	−20	3.86	62	4	−26	6.83					64	−10	−28	6.39
	LH	−58	−4	−34	4.21	−64	−14	−24	7.4					−62	−16	−18	8.29
middle temporal, anterior	RH													62	0	−24	5.81
	LH													−64	−8	−26	4.82
Superior temporal gyrus posterior	RH					68	−28	2	3.51								
	LH	−60	−36	0	3.85	−66	−32	2	4.65								
Superior temporal gyrus anterior	RH					52	−2	−16	3.85								
	LH					−56	−2	−12	4.5								
inferior parietal cortex	RH	54	−58	16	4.04	60	−62	24	4.62					48	−62	28	5.23
	LH	−52	−52	20	3.21	−62	−60	26	5.42					−40	−74	36	6.88
posterior insula	RH	40	−12	6	3.04	36	−14	12	4.87					38	−20	4	3.47
	LH	−40	−10	−2	3.33	−40	−18	0	3.65					−38	−6	−12	4.01
parahippocampal gyrus	RH	28	−12	−32	4.79	30	−16	−28	3.9					18	−8	−28	4.8
	LH	−32	−38	−12	3.49	−32	−30	−18	7.28					−24	−16	−28	6.23
postcentral gyrus	RH	16	−34	76	3.35	46	−26	64	3.51					22	−42	62	5
	LH	−20	−44	66	3.06	−20	−46	70	4.42					−64	−16	16	4
Precentral gyrus	RH													12	−26	44	3.67
	LH													−4	−24	58	3.29
caudate	RH													18	4	22	3
	LH													−14	−2	18	3.03
Cerebellum Crus II	RH					22	−82	−42	3.07					26	−82	−46	3.16
Occipital pole	RH	34	−92	22	4.5	36	−92	−4	4.57	38	−92	12	9.21	34	−92	−6	10.6
	LH	−32	−92	26	4.71	−34	−90	8	5.4	−36	−94	8	6.01	−16	−92	0	7.87
inferior occipital gyrus	RH	36	−82	−16	3.3	42	−82	−4	5.96	50	−70	−14	4.46	34	−88	0	6.85
	LH	−36	−84	−12	3.34	−34	−82	−14	5.87	−40	−90	−10	5.35	−44	−80	−6	5.86
fusiform gyrus (FFA)	RH	34	−54	−18	4.02	30	−52	−16	5.62	30	−44	−20	5.25	30	−60	−10	5.72
	LH	−28	−62	−14	3.05	−30	−58	−18	5.23	−32	−54	−14	5.46	−30	−62	−20	6.3
Lateral occipital cortex	RH	38	−92	12	4.26	40	−82	4	6.07	40	−86	18	7.92	38	−86	12	5.62
	LH	−38	−86	−8	3.41	−38	−80	−2	7.19	−40	−90	16	7.98	−46	−82	−6	5.71
Inferior temporal, posterior	RH	54	−60	−14	3.53	54	−60	−12	7.83	58	−54	−16	5.16	56	−30	−22	2.95
	LH					−48	−54	−20	6.29	−56	−42	−28	2.99	−44	−62	−22	6.29
superior parietal lobule	RH	26	−70	46	6.9	30	−44	46	7.07	24	−76	40	5.29	26	−76	38	8.67
	LH	−32	−46	42	5	−32	−44	42	6.61	−22	−84	38	5.93	−28	−46	42	8.88
frontal eye-fields	RH	26	4	64	3.08	34	−2	54	4.96	34	6	60	3.1	28	4	62	7.44
precentral gyrus	RH	50	2	28	4.43	54	8	8	5	52	6	36	3.24	44	0	38	5.16
	LH	−42	−4	40	3.4	−44	−2	32	7.97	−38	−2	36	4.65	−50	2	38	7.82
Inferior frontal gyrus, pars opercularis	RH	48	8	20	5.14	56	14	4	5.23	36	24	8	6.45	52	16	2	7.14
	LH	−52	22	20	3.65	−34	10	26	4.91	−46	12	10	3.39	−50	20	34	4.6
Inferior frontal gyrus, pars triangularis	RH	54	36	8	3.08					52	28	4	3.11	54	24	20	5.05
	LH	−50	32	20	4	−40	26	22	4.95	−48	24	−2	3.6				
anterior insula	RH	30	26	−6	4.38	36	18	−6	6.4	32	18	−2	5.44	38	20	0	8.58
	LH	−26	24	−4	3.63	−40	18	0	4.96	−38	18	0	4.38	−32	16	2	8.51
paracingulate/anterior cingulate	RH	4	18	48	3.65	4	20	54	6.95	10	26	44	5.22	12	26	26	5.22
	LH	−2	14	46	3.7	−2	16	52	6.36	−8	34	36	5.39	−10	22	30	5.8
dorsolateral prefrontal cortex	RH	48	30	32	4.03	48	28	30	4.89								
	LH	−50	32	20	4	−46	34	30	6.06	−40	0	48	3.83	−48	30	22	7.04
anterior/lateral thalamus	RH					10	−14	6	4.68	18	−24	2	3.42	8	−16	12	3.19
	LH					−14	−20	8	3.11	−12	−16	2	2.69				
pallidum	RH					18	0	−4	3.8					18	−12	−2	3.14
	LH													−14	−4	−6	3.2
putamen	LH									−14	10	−4	3.22				
cerebellum Crus I	RH	40	−64	−26	3.78	40	−56	−36	3.38	50	−46	−40	2.73				
	LH	−28	−64	−36	3	−42	−66	−34	6.11					−30	−74	−28	7.06
cerebellum Crus II	RH									36	−60	−44	2.57				
	LH									−6	−74	−34	2.6	−4	−76	−34	6.96
cerebellum VI	LH									−8	−70	−28	2.82	−34	−64	−24	5.61
cerebellum VIIb	RH									36	−62	−52	3.42				
	LH					−6	−76	−44	7.55	−10	−72	−46	3.1	−6	−74	−46	5.87
cerebellum VIIIa	RH									−22	−68	−58	3.18	28	−70	−60	5.77
cerebellum vermis VIIIb	RH					18	−50	−52	2.82								
	LH					−4	−60	−40	5.59								

#### Attending to the mouth (see figures 3 and 4, right panels, table 2)

Attending to the mouth in upright faces resulted in comparable patterns of activation for NT as observed when attending to the eyes *(see activation in yellow)* whereas ASD exhibited no activation in this condition. For inverted faces, patterns of activation for both groups were comparable to the activation observed when they were cued to the eyes *(see activation in blue)*.

### Between-group Whole Brain Activation Analyses

#### Attending to the eyes (see table 3, figure 5)

For upright faces, ASD showed increased activation compared to controls in the thalamus, the caudate, and at a more liberal threshold (*p*<0.01) in the superior colliculus. No area showed more activation in NT vs. ASD for upright faces. For inverted faces, NT exhibited more activation in several areas including the IFG, the anterior insula, anterior cingulate, pallidum, prefrontal cortex and cerebellum. ASD did not show increased activation in any area compared to NT when attending to the eyes in inverted faces.

**Figure 5:**
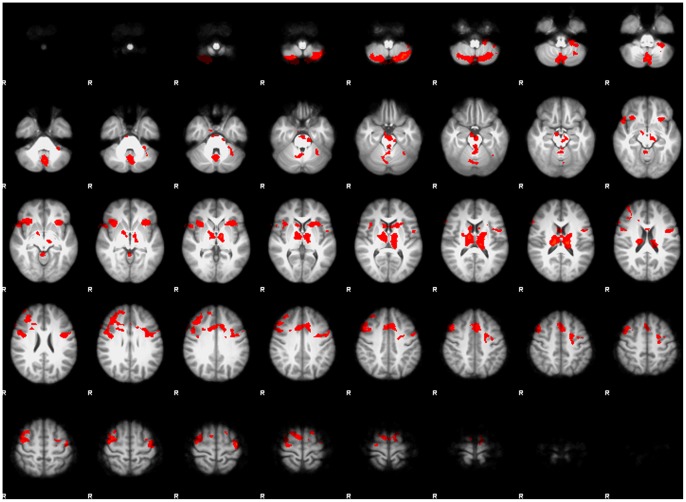
Between-group statistical map for the upright vs. inverted eye-cued condition (Z>2.3, corrected cluster significance of *p* = 0.05). This map shows brain regions that are significantly different between groups. To see whether the difference is due to ASD>NT or NT>ASD, refer to Table 3.

**Table 3:** Between-group contrasts when participants are attending to the eyes.

		AREA	X; Y; Z	Zscore
**EYES UP>INV**	**NT>ASD**	none		
	**ASD>NT**	Caudate	8; 10; 12	3.40
		Caudate	−18; 20; 4	3.16
		Left thalamus	−14; −28; 14	4.25
**EYES INV>UP**	**NT>ASD**	Inferior frontal gyrus, pars opercularis	54;16; −6	3.32
		Anterior insula	36; 18; 0	3.53
		Anterior insula	−32;22; −4	3.39
		Pallidum	16; 2; −4	3.11
		Middle cingulate	10; 18; 38	3.19
		Middle cingulate	−4; 22; 42	3.38
		Precentral gyrus	46; −4;60	3.41
		Precentral gyrus	−44; −10; 64	3.92
		Dorso-lateral prefrontal cortex	40; 32; 30	3.23
		Middle frontal gyrus	38;2; 60	3.77
		Superior frontal gyrus	8; 0; 70	3.43
		Superior frontal gyrus	−20; 14; 68	3.45
		Supplementary motor area	8; 6; 68	3.23
		Right thalamus	18; −24; 14	3.95
		Cerebellum I–IV	2; −46; −6	3.93
		Cerebellum I–IV	0; −48; −22	3.16
		Cerebellum VIIIa	20; −66; −52	3.67
		Cerebellum VIIIa	−20; −64; −25	3.18
		Cerebellum vermis VIIIa	−2; −68; −42	3.67
		Cerebellum VIIb	26; −68; −52	3.22
		Cerebellum VIIb	−28; −68; −56	3.67
		Cerebellum vermis IX	2; −56; −32	3.14
		Cerebellum VIIIb	−24; −40; −46	3.08
		Cerebellum VI	−32; −52; −30	3.06
	**ASD>NT**	none		

Z>2.3, cluster corrected *p* = 0.05.

#### Attending to the mouth

There were no significant differences between groups when participants were attending to the mouth, both for the upright and the inverted conditions.

At a more liberal threshold (*p*<0.001), NT showed higher activation in a large set of brain areas for upright faces, including areas associated with emotion processing (amygdala, orbitofrontal cortex) and mentalizing (mPFC, posterior cingulate/precuneus, temporal pole). There were no areas for which ASD showed increased activation compared to NT when attending to the mouth in upright faces. For inverted faces, NT exhibited more activation than ASD in the anterior insula, visual cortex, IFG (pars opercularis) and cerebellum (Crus I, VI, VIIIa), while the ASD group showed increased activation in the inferior lateral occipital cortex. IFG (pars triangularis) and superior temporal gyrus were significantly different between groups, with NT showing increased activation for upright faces and ASD for inverted faces.

#### A priori ROI analysis (See figures 6 and 7)

**Figure 6:**
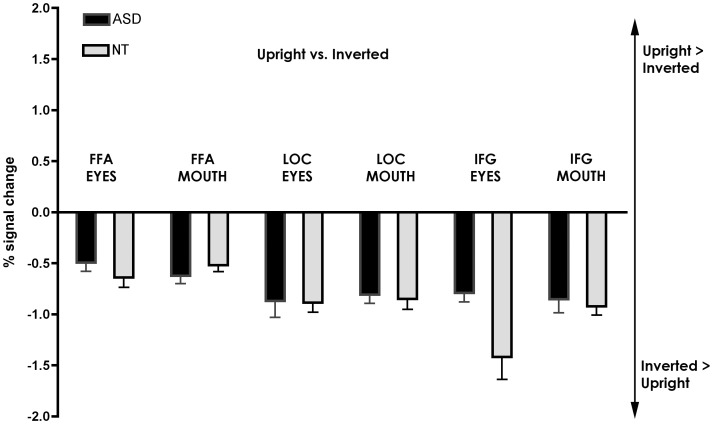
Region of interest analysis. Percent BOLD signal change with standard errors for the contrast upright>inverted in cortical areas, including the right FFA, LOC and IFG. Negative values show that inverted faces led to significantly more activation than upright faces in those brain areas.

For the cortical ROIs, the FFA, LOC and IFG, activation was significantly different from zero when comparing upright vs. inverted presentation in both groups and in both feature conditions, with increased activation observed for the inverted orientation (all *t*>5.33, *p*<0.001). For the FFA and LOC, ANOVAs revealed no main effect of Group, Feature, or Feature x Group interaction (all *F*<3.1) indicating that ASD showed similar activation than NT in face and object areas. In contrast, a significant Feature x Group interaction was found for the IFG (*F*(1.32) = 5.09, *p*<0.05) due to increased activation in NT, specifically when cued to the eyes (*p*<0.01) (See Figure 6).

**Figure 7:**
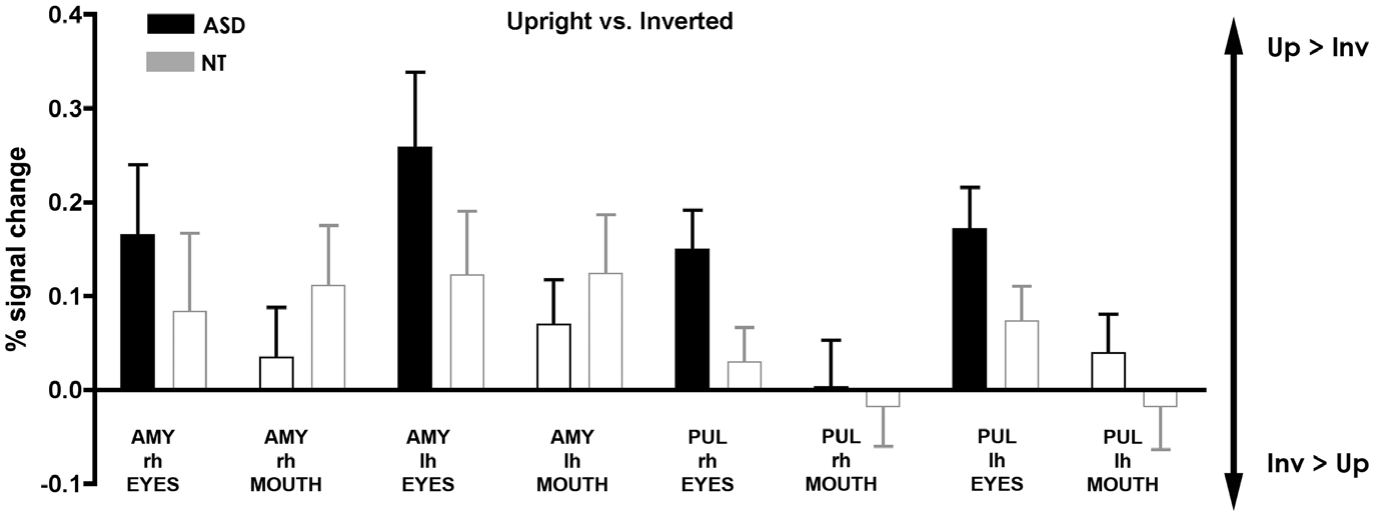
Region of interest analysis. Percent BOLD signal change with standard errors for the contrast upright>inverted in subcortical areas including the amygdala and the pulvinar for the right hemisphere (rh) and the left hemisphere (lh). Areas that were significantly different across Orientation (upright vs. inverted) are represented in solid color, and only the contours of those that failed to reach significance are shown.

Results for the subcortical ROIs are shown in Figure 7. One sample *t*-tests against zero conducted to assess differences between orientation revealed a significant activation in ASD for AMY and PUL in both hemispheres, indicating that those areas showed increased activation for upright faces in the eye-cued condition (all *t*(15)>2.22, *p*<0.05). There was however no significant activation in the mouth-cued condition. For NT, no significant effect was found in either structure for either condition.

## Discussion

Using a Thatcher Illusion paradigm, we demonstrated that when individuals with ASD were cued to attend to the eye-region (as opposed to the mouth) in upright faces, they showed increased face discrimination accuracy, enhanced activation in cortical areas involved in social and emotional processing and concurrent hyper-activation in subcortical areas.

### Configural Processing and Importance of the Eyes, Evidence from Behavioral Data

The TI is one of the experimental paradigms allowing assessment of configural face processing. Consistent with previous findings [34], [49], the behavioral data revealed that individuals with ASD as well as NT are sensitive to the TI, as illustrated by significantly decreased performance for discriminating between a thacherized and a typical face when presented inverted as opposed to upright, and the absence of a Group x Orientation interaction.

One of the behavioral marker for a loss of configural processing in faces is a reduced face inversion effect (FIE): the FIE is defined by the reduction in performance for inverted face recognition and identity matching relative to upright faces [8], [50], [51], [52], [53]. Initial studies have reported a reduced FIE in individuals with ASD (e.g. [54]). However, further studies have reported normal FIE in this population (e.g. [26], [43], [55]). Our data add to the body of literature suggesting that impairments in face processing in ASD are not due to a generalized configural processing deficit (reviewed in [11], [27]). Individuals with ASD were however generally less accurate than NT in recognizing thatcherized stimuli, independent of feature and orientation, supporting the hypothesis of difficulties in face processing. The significant interaction of Feature x Orientation x Group found in the current study for error rates resulted from the fact that NT were particularly impaired at discriminating the two faces during the single feature mouth condition in inverted faces. The accuracy of the NT did not differ across single features in the upright condition, due to a performance close to ceiling, but differed for the inverted condition, with cueing to the mouth rendering the task more difficult (See Figure 2). It has been shown that less salient facial regions such as the mouth are more affected by face inversion [56]. On the other hand, individuals with ASD showed better performance when cued to the eyes compared to the mouth in upright faces, and a trend for better discrimination when cued to the eyes compared to mouth in inverted faces. High error rates for the inverted condition were due to higher number of omissions for this orientation. Reaction times did no differ between groups for the upright orientation, however for inverted faces, ASD showed faster reaction times, but did not make fewer errors than NT, suggesting they guessed the answer when the discrimination became particularly difficult.

In conclusion, our behavioral data confirm that individuals with ASD are sensitive to the TI, supporting the presence of configural processing. In addition, they show that directing visual attention towards the eyes, the most salient feature in typical face processing [57] and key in driving the TI, leads to better face discrimination ability in ASD.

Experiments using the Thatcher Illusion have shown deficits in configural face processing along with preserved featural processing in individuals with prosopagnosia, a disorder characterized by severe impairments in recognizing familiar faces [18], [19]. However, while both individuals with ASD and individuals with prosopagnosia exhibit impairments in processing information from faces, the underlying causes are of very different nature. While prosopagnosia is essentially a disorder of face identification, linked with abnormal function of the FFA and/or occipital face area [36], [58], [59], face-processing difficulties in ASD are on the other hand mainly associated with deficits in emotional processing, possibly linked to reduced motivation to attend to social stimuli [60].

### Enhancement of Social and Emotional Processing by Cueing to Eyes

Activation maps showed that an extensive network of areas involved in social and emotional processing was activated by the discrimination of upright thatcherized faces in both groups. Discrimination of upright faces while attending to the eye-region is the condition for which ASD and NT groups showed the least functional difference. Notably, whole brain analysis showed a similar increase in mPFC and posterior cingulate/precuneus activation for upright grotesque face discrimination while attending to the eyes in both groups. These regions have been implicated in emotional processing, including attribution of emotion/mentalizing [21], [61], [62], [63], [64], [65]. The mPFC has a role in top down biasing towards treating information as socially relevant [66]. This underlines the fact that if the paradigm requires participants to attend to the eye-region in upright faces, brain activation in areas associated with social processing can be alike in ASD and NT groups. However, when participants were cued to mouths in upright faces, the NT group alone showed activation in the mPFC and the posterior cingulate cortex/precuneus cortex at a more liberal threshold (*p*<0.001). Most of our cognition occurs automatically and without awareness [66]. We speculate that activation in mPFC and posterior cingulate cortex/precuneus cortex could be due to a spontaneous orienting of NT to the eyes, when cued to the mouth, reflecting typical attention to the most salient region of the face, the eye region. Several studies have indeed demonstrated that NT point of regard naturally gravitates to the eyes [67], [68]. We suggest that the lack of activation in the aforementioned areas in ASD is due to the fact that ASD, in contrast to NT, strictly follow the cueing instructions and perform the discrimination without implicit emotional processing induced by gazing to the eye-region. Our current findings are however limited by the fact that we did not collect eye-tracking data during fMRI image acquisition, and future eye-tracking studies should help clarifying this point. Amygdala activation correlates with time spent looking in the eye region of the face [69]. Supporting the notion that NT spontaneously re-orient towards the eye region, increased amygdala activation was observed in the mouth-cued condition in NT. In line with this, a recent combined fMRI eye-tracking study reported increased amygdala activation when typicals as opposed to ASD first looked at the mouth reflecting increased re-orientation to the eye region in typicals [29]. Furthermore, previous research has shown that typically developing children cannot resist an uninformative gaze cue in attention paradigms, which is not the case in children with ASD [70].

### Face Processing Network

Face processing involves a distributed network of cortical and subcortical areas, including the inferior occipital gyrus, the FFA, the superior temporal sulcus, the insula, the IFG, the amygdala, and pulvinar (e.g. [71], [72], [73], [74]). There has been a long controversy about the involvement of the FFA in ASD. Initial studies that did not control for gaze patterns reported reduced activation in this region (e.g. [75], [76]), but subsequently others have suggested that this reduced activation may originate in atypical eye-gaze patterns towards faces. These more recent studies indicate that FFA activation depends on orientation towards the eyes during stimulus presentation both in neurotypicals [69] and in individuals with ASD [4], [28].

In the current study, discrimination of thatcherized stimuli led to increased activation of an extended face-processing network in both ASD and NT for inverted faces. First, it is important to note that in inverted thatcherized faces, the eyes or mouth are in fact upright, given that thatcherization consisted in inverting those regions in upright faces. Additionally, this increased activation in inverted faces could also be due to a greater workload allocation in order to perform the task in this orientation, and possibly also due to the fact that thatcherized faces are less ecologically face-like in their upright than in their inverted orientation. Face inversion was also shown to lead to increased latency and amplitude of the N170, an electrophysiological response sensitive to faces [77], [78], [79]. It is important to note that no between-group differences were observed in the FFA, and that both ASD and NT showed increased activation for the discrimination of inverted thatcherized faces.

The significantly decreased activation of the IFG when participants with ASD were cued to eyes, compared to the activation seen in NT, is in line with previous studies reporting decreased activation of the IFG during face processing in ASD. This finding is relevant for a mirror neuron system hypo-activation theory in ASD [80], [81]. Additional areas in which participants with ASD showed decreased activation compared to NT included the anterior insula and the cerebellum. The anterior insula is involved in the evaluation of task performance as well as in social and emotional processing; hypoactivation of this region in individuals with ASD is consistent with the findings from neuroimaging studies using social stimuli [82]. The role of the cerebellum in cognitive processing is still poorly understood. Here, differences in the cerebellum were systematically found between the ASD and NT groups for inverted face processing, in areas known to be functionally connected with motor and cognitive association areas [83]. The findings indicate that the role of cerebellum in face processing in individuals with ASD requires further investigation.

### Subcortical System

The superior colliculus, the pulvinar nucleus of the thalamus and the amygdala are key elements of the subcortical face-processing pathway [74], [84]. Specifically for the condition in which they were cued to eyes in upright faces, individuals with ASD showed increased activation compared to NT individuals in these subcortical areas.

The development of eye contact seems to be disrupted in ASD, although apparently contradictory results have been reported, with some showing stronger neurophysiological response to direct gaze [85], [86], [87], [88], while others showed no such effect [89], [90]. Previous research has suggested that a global face-configuration in newborns activates the subcortical system as a means to orient towards faces, a phenomenon known as CONSPEC [91]. CONSPEC may also be the mechanism underlying eye-contact detection [92], [93] that leads to the preference for the eye region seen in NT individuals during face processing, and seemingly absent in individuals with ASD. Expert face processing builds on the maturation of other circuits devoted to face processing, which require sufficient opportunity to process faces and depends on motivation and/or social orienting mechanisms. The subcortical system remains active in neurotypical adults during emotional face processing, allowing rapid orienting towards biologically-relevant stimuli [84], [94], [95], [96], [97], [98]. In the current study, we saw a greater engagement of the subcortical route for discrimination of grotesque faces in individuals with ASD when cued to look at the eye-region in upright faces. We suggest that this effect may be due to an emotional response induced by looking at the eye-region, possibly resulting from an immature or hypersensitive subcortical system. Increased activation of the subcortical route, a system normally engaged in emotional processing and location of threat in our environment [99], may lead to a mistaken interpretation of threat during face perception that underpins active disengagement from faces, especially from the eye-region in individuals with ASD.

Our data suggest abnormal involvement of the subcortical route during complex face discrimination in the ASD group. Further studies should address the neural substrates of eye-contact aversion in individuals with ASD, and test whether an alteration in face-detection systems can provide a theoretical account of a behavior that jeopardizes smooth social interactions.

### Conclusions

In conclusion, our data indicate that individuals with ASD are sensitive to the TI, supporting the presence of configural face processing. We observed large group similarities in the face-processing network in response to inverted thatcherized faces. Our results show that directing visual attention towards the eyes in upright faces leads to better behavioral performance and to increased activation in cortical areas involved in emotional and social processing.

Our data also indicate a heightened activation of subcortical areas in ASD when their attention is directed towards the eyes. This observation suggests a mechanism by which over-activity in the subcortical system could lead to unpleasant arousal and active eye-avoidance in people with ASD.

Given the ample evidence of difficulties in eye-discrimination in ASD, one key question has been whether a deficit in face-processing leads to a deficit in social attention, or whether it is the consequence of the latter [27]. Our findings indicate that face-processing, including its social and emotional aspects, may be enhanced in ASD when social attention is warranted by explicit cueing [28]. Our results may also have implications for behavioral therapies aimed at improving face processing. If social attentional processes underlie face-processing difficulties, then, to ensure improvement that generalizes to all aspects of face-processing, explicit cueing to the eyes should be a crucial component of the training.
